# Iron Fortification of Lentil (*Lens culinaris* Medik.) to Address Iron Deficiency

**DOI:** 10.3390/nu9080863

**Published:** 2017-08-11

**Authors:** Rajib Podder, Bunyamin Tar’an, Robert T. Tyler, Carol J. Henry, Diane M. DellaValle, Albert Vandenberg

**Affiliations:** 1 Department of Plant Sciences, University of Saskatchewan, Saskatoon, SK S7N 5A8, Canada; rap039@mail.usask.ca (R.P.); bunyamin.taran@usask.ca (B.T.); 2 Department of Food and Bioproduct Sciences, University of Saskatchewan, Saskatoon, SK S7N 5A8, Canada; bob.tyler@usask.ca; 3 College of Pharmacy and Nutrition, University of Saskatchewan, Saskatoon, SK S7N 5C9, Canada; carol.henry@usask.ca; 4 Department of Nutrition and Dietetics, Marywood University, 2300, Adams Avenue, Scranton, PA 18509, USA; ddellavalle@maryu.marywood.edu

**Keywords:** lentil, iron, fortification, NaFeEDTA, FeSO_4_·7H_2_O, FeSO_4_·H_2_O

## Abstract

Iron (Fe) deficiency is a major human health concern in areas of the world in which diets are often Fe deficient. In the current study, we aimed to identify appropriate methods and optimal dosage for Fe fortification of lentil (*Lens culinaris* Medik.) dal with FeSO_4_·7H_2_O (ferrous sulphate hepta-hydrate), NaFeEDTA (ethylenediaminetetraacetic acid iron (III) sodium salt) and FeSO_4_·H_2_O (ferrous sulphate mono-hydrate). We used a colorimetric method to determine the appearance of the dal fortified with fortificants at different Fe concentrations and under different storage conditions. Relative Fe bioavailability was assessed using an in vitro cell culture bioassay. We found that NaFeEDTA was the most suitable fortificant for red lentil dal, and at 1600 ppm, NaFeEDTA provides 13–14 mg of additional Fe per 100 g of dal. Lentil dal sprayed with fortificant solutions, followed by shaking and drying at 75 °C, performed best with respect to drying time and color change. Total Fe and phytic acid concentrations differed significantly between cooked unfortified and fortified lentil, ranging from 68.7 to 238.5 ppm and 7.2 to 8.0 mg g^−1^, respectively. The relative Fe bioavailability of cooked fortified lentil was increased by 32.2–36.6% compared to unfortified cooked lentil. We conclude that fortification of lentil dal is effective and could provide significant health benefits to dal-consuming populations vulnerable to Fe deficiency.

## 1. Introduction

Lentil (*Lens culinaris* Medikus) is an important legume crop, cultivated for food and feed since prehistoric times. As a source of dietary protein, lentil can be combined with cereals to prepare human diets and animal feeds that provide a balance of essential amino acids and essential micronutrients such as iron, zinc and selenium [1,2]. Lentil is a good source of non-heme iron, ranging from 73 to 90 mg kg^−1^ [3]. The crude protein content (*N* × 6.25) of Western Canadian lentil is reported to range from 25.8 to 27.1% [4]. Lentil also is considered to be a starchy legume as it contains 27.4–47.1% starch, with a significant level of amylose (23.5–32.2)% [5,6]. Although lentil is a good source of intrinsic Fe, the bioavailability/absorption is low [7]. These authors reported that the mean Fe absorption from lentil dal was 2.2%, which was significantly lower than the 23.6% observed for a similar amount of Fe given as ferrous sulphate to women with poor Fe status. Low bioavailability may be due to the presence of phytic acid and polyphenols in the lentil dal [7,8].

Iron (Fe) is the most abundant element in the earth′s crust and is an essential micronutrient for both plants and animals. In plants, Fe deficiency affects key metabolic processes such as the electron transfer system for photosynthesis and respiration [9]. Iron deficiency in humans refers to a condition in which an insufficient amount of bioavailable Fe results in Fe deficiency anemia [10]. This deficiency has become a major nutritional disorder, widespread in both developing and developed countries [11]. The major consequences of Fe deficiency are reduction of physical activity, fitness and work capability, a reduced ability to maintain body temperature, a lowered resistance to infection, and an increase in mortality during pregnancy and in newborns [12]. According to Food and Agriculture Organization (FAO) and World Health Organization (WHO) recommendations, the estimated daily average Fe requirements for females and males 19–50 years of age are 29.4 mg and 10.8 mg, respectively, based on 10% bioavailability [13].

Several strategies are used around the world to address micronutrient malnutrition. Micronutrient supplementation, dietary diversification, biofortification, food fortification, nutrition education, public health interventions and food safety measures are approaches that can solely or in combination be applied to address micronutrient deficiency in a target population [14]. Supplementation is an effective means of providing immediate benefits to “at risk groups” but not for other household or community members [15] since it requires supplemental Fe consumption on a long-term basis, in tablet form for example. Dietary improvement through supplementation requires a change in dietary behavior, and this process also requires changes in food supply and availability that may require a long time to achieve success [14]. Also, public health intervention can help prevent micronutrient malnutrition, but micronutrient malnutrition can also be associated with a high prevalence of microbial infection that causes a variety of different diseases. Food fortification can overcome this limitation due to its sustainability in improving the dietary quality of a targeted group or population without changing dietary habits. Food fortification is a potentially cost-effective way to add micronutrients to processed foods in a way that can rapidly mitigate micronutrient malnutrition [13].

A successful Fe fortification program was first reported in Canada in 1944, when the government began fortifying wheat flour with Fe along with thiamine, riboflavin and niacin [14]. A remarkable reduction in child mortality was observed from 102/1000 live births in 1944 (first year) to 61/1000 in 1947 in Canada [16]. During the twentieth century, Fe fortification became mandatory in several developing countries, including Bolivia, Chile, Colombia, Costa Rica, Ecuador, Guatemala, Indonesia and others [17]. In every country, either wheat or maize flour was chosen as the food vehicle. The requirements for selecting an appropriate food vehicle for fortification were established by FAO in 1995 [17]. In 1980, the FDA (U.S. Food and Drug Administration) established a “Food Fortification Policy” that was guided by six basic principles [18]. The WHO has recommended Fe compounds and concentration for fortification of wheat flour in 13 countries [19]. To optimize iron bioavailability and maintain the organoleptic attributes that influence consumer acceptability of fortified foods, selected food vehicles and Fe fortificants need to be well matched. The food vehicle should be safe, widely accepted by the target consumers, have good storage capability after fortification, and the added Fe should be stable with high bioavailability [20].

Fortifying lentil with suitable Fe fortificants is a research area with potential application to reduce Fe deficiency. We hypothesized that it would be possible to increase the amount of bioavailable Fe in dehulled (decorticated) pulses (dal) such as lentil, in a biologically and culturally meaningful way, to a level that could prevent Fe deficiency in humans. Our experimental approach had two main objectives, first, to determine the most suitable iron fortificant and the appropriate dose of Fe for dehulled lentil based on ease of fortification, and second, to determine the optimal processing technology to fortify iron in dehulled lentil based on current processing practices. To fulfill the first objective, research was focused on selection of the appropriate genotype and product type of dehulled lentil, and identifying the best form of Fe solution with which to fortify dehulled lentil products. The Fe fortificants, ferrous sulphate heptahydrate (FeSO_4_·7H_2_O), NaFeEDTA (ethylenediaminetetraacetic acid iron (III) sodium salt) and ferrous sulphate monohydrate (FeSO_4_·H_2_O), are acceptable fortificants that have potential for fortifying dehulled lentil seed [13]. The second objective was fulfilled by conducting studies to help standardize the protocol for lentil fortification. These included assessments of the appropriate dose of Fe solution, selection of the most appropriate fortification method in the context of changes in organoleptic properties and storage capability, assessment of the best temperature for drying lentil after the addition of fortificants, and the effect of fortification on boiling time.

## 2. Materials and Methods

**Figure 1:**
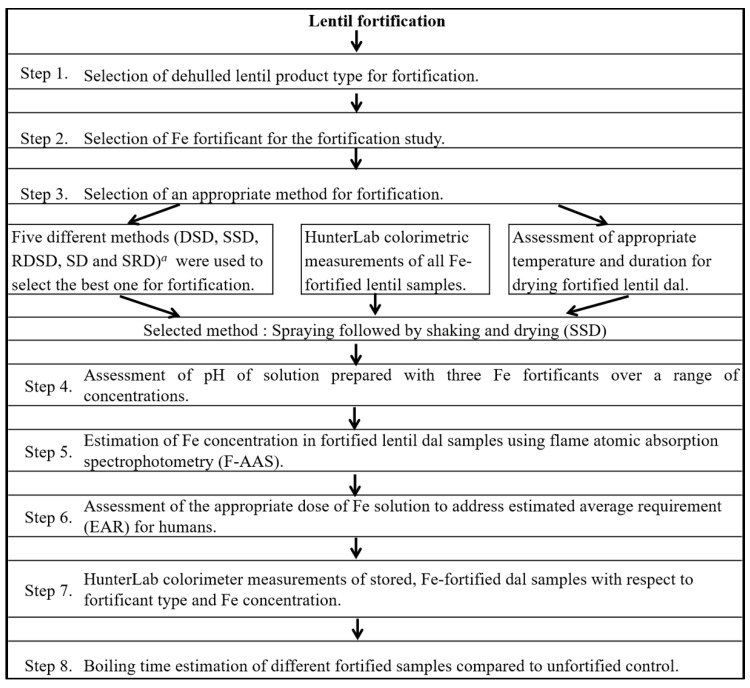
Flow chart for development of a lentil fortification protocol. *^a^* Oven dried, soaked and oven dried (DSD); sprayed followed by shaking and drying (SSD); rinsed, oven dried, soaked, and oven dried (RDSD); directly soaked in Fe solution (SD) and rinsed, soaked, and oven dried (SRD).

The procedure followed for development of a lentil fortification protocol is shown in Figure 1, and is discussed below.

### 2.1. Selection of Lentil Genotype and Dehulled Lentil Product Type

Fifteen red cotyledon lentil cultivars/genotypes were analyzed to estimate the concentration (ppm) of Fe in seeds (data not shown). One widely grown and popular cultivated red lentil cultivar, CDC (Crop Development Centre) Maxim, developed at the Crop Development Centre, University of Saskatchewan, Saskatoon, SK, Canada, was selected for fortification studies due to its having a high Fe concentration (75–90 ppm) compared to other red lentil cultivars grown in Saskatchewan [21].

**Figure 2:**
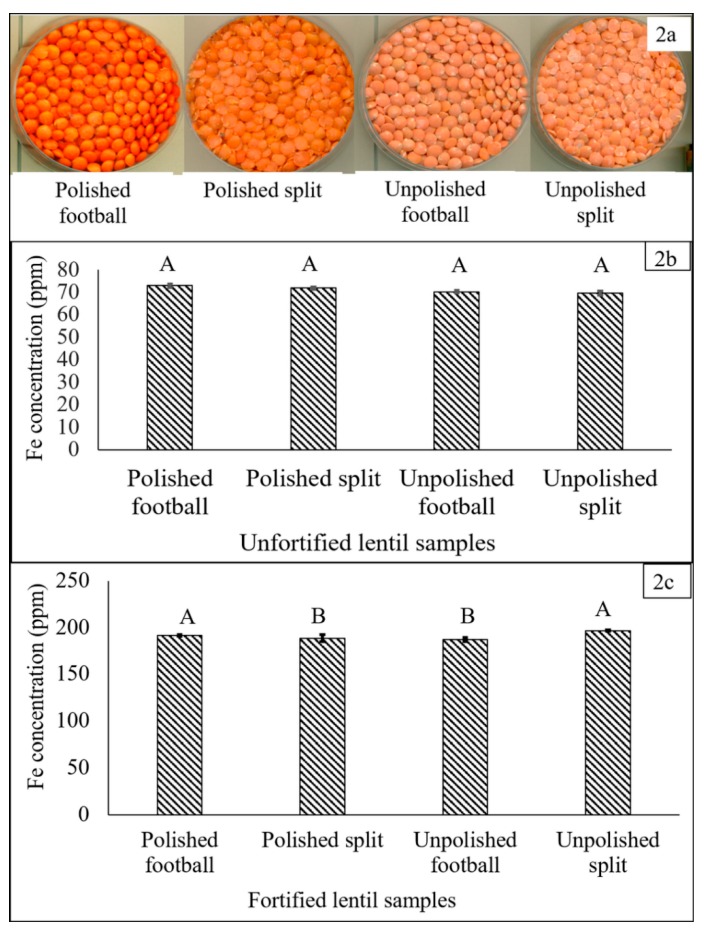
(**a**) Four dehulled, red lentil product types; (**b**) Fe concentration (ppm) in four dehulled, unfortified, red lentil product types; and (**c**) Fe concentration (ppm) in red lentil product types fortified with FeSO_4_·7H_2_O solution (400 ppm Fe). Different letters within each figure represent significant differences (*p* < 0.05).

Four different types of dehulled lentil products are usually available in the red lentil market: polished football (dehulled, unsplit), polished splits, unpolished football and unpolished splits (Figure 2a). The Fe concentration in each product type was measured to determine the range of variability in Fe concentration. The product types then were used in a fortification study and samples of 200 g of each product type were mixed with 20 mL of NaFeEDTA solution (1600 ppm Fe) with four replications. The best product type in relation to uniformity of absorption of Fe solution, drying time and concentration of Fe in the fortified product was selected. The statistical analysis was conducted using SAS version 9.4 (SAS Inc., Cary, NC, USA). One-way analysis of variance (ANOVA) was used to compare the Fe concentration of unfortified and fortified red lentil product types. The least significant difference (LSD) was calculated and the level of significance set at *p* < 0.05.

### 2.2. Selection and Evaluation of the Most Suitable Fe Fortificant for Lentil

The selection of the most appropriate Fe fortificant is challenging due to possible interactions between the food product and the Fe compound. Three water-soluble Fe compounds, FeSO_4_·7H_2_O, NaFeEDTA and FeSO_4_·H_2_O were selected from a list of iron fortificants published in the WHO and FAO document “Guidelines on Food Fortification with Micronutrients” [13]. The FeSO_4_·7H_2_O and FeSO_4_·H_2_O were supplied by Crown Technology, Inc., Indianapolis, IN, USA, and NaFeEDTA by Akzo Nobel Functional Chemicals, LLC, Chicago, IL, USA. The three fortificants were food grade and were selected on the basis of their relative bioavailability, interaction with the food vehicle and cost of fortification [14].

### 2.3. Selection of an Appropriate Method of Fortification

#### 2.3.1. Techniques Used for Lentil Fortification

An experiment was designed to determine the most appropriate method for fortifying dehulled, polished, football lentil dal with an Fe solution prepared with FeSO_4_·7H_2_O, one of the three Fe fortificants studied. Five methods were used to fortify lentil dal with FeSO_4_·7H_2_O solution (1600 ppm Fe) at 10 mL fortificant solution/100g dal. The 1600 ppm Fe concentration was selected with the aim that this concentration may provide a major part of the recommended daily allowances (RDAs) for humans. However, each method to fortify lentil dal is described below.

*Method 1 (Dry-Soak-Dry).* Lentil dal was oven dried at 80 °C for 10 min, soaked in 10 mL of fortificant solution for 2 min, and then dried again at 80 °C to obtain a moisture content of 14%.

*Method 2 (Spray-Shake-Dry).* Lentil dal was sprayed with fortificant solution using a 473 mL clear, fine-mist spray bottle (SOFT ′N STYLE, Product Code VO-302564, SKS Bottle and Packaging, INC., Watervliet, NY, USA), shaken using a Barnstead Thermolyne M49235 Bigger Bill Orbital Shaker (Sigma-Aldrich Corp., St. Louis, MO, USA) at 400 rpm for 10 min to mix the solution with the dal sample, and subsequently dried to 14% moisture under a 250-watt electric heat lamp (NOMA incandescent, clear, 130 V heat lamp, Trileaf Distributors, Toronto, ON, Canada) which produced a temperature of approximately 70 °C at the surface of the fortified dal.

*Method 3 (Rinse-Dry-Soak-Dry).* The third method consisted of rinsing 100 g dal samples under a continuous flow of deionized water for 30 s followed by oven drying at 80 °C for 10 min. The dried sample then was soaked in the fortificant solution (10 mL fortificant solution/100 g lentil) for 2 min and then placed in the oven again for 15 min at 80 °C to reduce the moisture level to 14%.

*Method 4 (Soak-Dry).* Lentil dal was soaked in fortificant solution followed by oven drying at 80 °C to 14% moisture.

*Method 5 (Soak-Rinse-Dry).* Lentil dal was soaked in fortificant solution and then rinsed with deionized water for 30 s, followed by oven drying at 80 °C to 14% moisture.

#### 2.3.2. HunterLab Colorimetric Measurements of Fe-Fortified Lentil Samples

The color of the Fe-fortified lentil sample from each of the five fortification methods was measured using a HunterLab instrument (Hunter Associates Laboratory Inc., Reston, VA, USA) to allow comparison with unfortified control samples. For each method, four samples were assessed. The dimensions L*, a* and b* were compared with those of the control sample, where L* indicates lightness (ranging from 0 to 100), a* indicates red (+) and green (−) and b* indicates yellow (+) and blue (−) with a range of +80 to −80 [22]. The L*, a* and b* values were analyzed using ANOVA in SAS 9.4.

#### 2.3.3. Assessment of Appropriate Temperature and Duration for Drying Fortified Lentil Dal 

Electric heat lamps of three power levels (100, 200 and 250 watts) (Trileaf Distributor) were used to dry fortified football dal after spraying with fortificant solution. The distance between the bulb and the lentil dal surface was 15 cm. Samples of 100 g of dal were fortified with 10 mL of FeSO_4_·7H_2_O solution (1600 ppm Fe concentration). The maximum temperature (°C) in the middle of the fortified dal sample during drying with the three bulb types and shaking using a Barnstead Thermolyne M49235 Bigger Bill Orbital Shaker (Sigma-Aldrich Corp.) was assessed using a thermometer (VWR Scientific, Chicago, IL, USA). The time to achieve 14% moisture for each sample was recorded for each treatment method. Both temperature and drying time were assessed three times and the mean temperature and drying time were calculated.

### 2.4. Estimation of Fe Concentration in Fortified Lentil Dal Samples by Flame Atomic Absorption Spectrophotometry (F-AAS)

The iron concentration in the fortified lentil dal was analyzed by flame atomic absorption spectrophotometry (F-AAS, Nova 300, Analytic Jena AG, Konrad-Zuse-Strasse, Neu-Ulm, Germany). Each sample was sub-sampled and 0.5 g was digested in a 30-mL digestion tube with HNO_3_-H_2_O_2_ using an automatic digester (Vulcan 84, Questron Technology, Ontario, CA, USA). All chemicals (nitric acid (70%), hydrogen peroxide (30%) and hydrochloric acid (37%)) used for digestion were of analytical grade. The digestion was repeated twice, with three technical replications per repeat. In the digestion chamber, a total of 72 samples were digested in each run, along with eight standards (yellow lentil laboratory check) and four blanks. Samples were first digested with HNO_3_ at 90 °C for 45 min, followed by addition of 5 mL of 30% H_2_O_2_ and then further digested for another 65 min. The solutions were then reduced with 3 mL of 6 M HCl, followed by heating at 90 °C for 5 min prior to cooling to room temperature. All sample solutions were then diluted with deionized water to a volume of 25 mL. Six mL of each of the digested samples was then used to determine the Fe concentration as described previously [23]. The Fe concentration values were analyzed using ANOVA in SAS 9.4 to determine differences for Fe concentration among the fortified lentil samples within each of the three fortificants at concentrations ranging from 100 to 3200 ppm. The LSD was calculated and the level of significance set at *p* < 0.001.

### 2.5. Assessment of the Appropriate Dose of Fe Solution

A total of 51 different solutions of the three fortificants (17 solutions of each fortificant with Fe concentrations of 100, 200, 400, 600, 800, 1000, 1200, 1400, 1600, 1800, 2000, 2200, 2400, 2600, 2800, 3000 and 3200 ppm) were prepared to fortify dehulled lentil dal samples. Ten mL of each fortificant solution at each Fe concentration was added to a 100-g dal sample and processed using the SSD (Spray–Shake–Dry) method described earlier. Twenty-five Fe solutions were prepared using the three Fe fortificants at eight concentrations (200, 400, 800, 1200, 1600, 2000, 2800 and 3200 ppm of Fe plus deionized water as the control) to assess the effect of increasing fortificant concentration on the pH of the solutions, which was measured three times for each solution using a pH meter (Oakton H_2_O proof BNC pH tester, Cole-Parmer Scientific Experts, Montreal, QC, Canada). Data were analyzed using SAS 9.4. 

### 2.6. HunterLab Colorimeter Measurements of Stored Fe-Fortified Dal Samples 

The initial color of Fe-fortified lentil dal samples was measured using a HunterLab (Hunter Associates Laboratory Inc., Reston, VA, USA) instrument. Twenty-seven samples (nine concentrations of each of the three Fe fortificants) and one control (unfortified lentil dal) with four replications were scored for their L*, a* and b* values. Samples of each treatment were stored individually at room temperature (25 °C) for one year in clear plastic bags (Ronco, Toronto, ON, Canada), similar to methods traditionally used to store dal products. After six months and one year of storage, the L*, a* and b* values of the lentil dal again were measured to determine if any color change had occurred. The one-year storage period was considered an approximate maximum storage period from processing to consumption by dal consumers. The L*, a* and b* values were analyzed using ANOVA in SAS 9.4.

### 2.7. Boiling Time Estimation of Fortified Lentil Dal Samples 

Three fortified dal samples (FeSO_4_·7H_2_0, NaFeEDTA and FeSO_4_·H_2_O at 1600 ppm Fe concentration) and one unfortified control were used to determine if differences existed in boiling time between fortified samples and the control. Two hundred fifty grams of each of the lentil dal samples were cooked in 1L of deionized water containing 5 g of NaCl on a single burner gas stove at 104 °C. The boiling time was recorded as the point when >90% of the dehulled lentils were softened to the point that the mixture with water produced a thickened soup, a method of preparation like that commonly used in the South Asian Region [24]. This study was replicated three times and data were analyzed using SAS 9.4. 

### 2.8. Relative Fe Bioavailability and Phytic Acid Content of Fortified Lentils

Lentil dishes were prepared for four different samples, including Fe-fortified lentil and the control (unfortified lentil). Both fortified and control samples were rinsed with 18 MΩ deionized water. A traditional Bangladeshi lentil dish (dal) was prepared in stainless steel cookware using a traditional Bangladeshi recipe [24] where salt, turmeric powder, onion, canola oil and deionized water were used as ingredients at a 15:75:5:3:2 ratio. The prepared dish was cooled to room temperature for 2 h, frozen at −80 °C for 24 h, freeze dried using a FreeZone 12 Liter Console Freeze Dry System with Stoppering Trays (Labconco, model 7759040, Kansas City, MO, USA) for 72 hand stored at room temperature [25]. Ten grams of freeze-dried dal from each dish was finely grounded and sent to the USDA-ARS Robert Holley Center for Agriculture and Health (Ithaca, New York, NY, USA) to assess iron concentration and bioavailability using an in vitro digestion/Caco-2 cell culture bioassay [26]. Total Fe concentration from the cooked lentil samples was measured using a standard HNO_3_-HClO_4_ method and atomic absorption spectrophotometry [23]. The phytic acid (total phosphorus) test kit (Megazyme International, County Wicklow, Ireland), a simple, quantitative, colorimetric and high throughput method [25,27], was used for the measurement and analysis of phytic acid in the four cooked lentil samples used for the bioavailability assessment. The ANOVA was conducted using SAS 9.4 to determine differences in iron concentration, relative iron bioavailability and phytic acid concentration among the cooked fortified lentil dishes. The LSD was calculated and the level of significance set at *p* < 0.001.

## 3. Results and Discussion

### 3.1. Selection of Dehulled Lentil Product Type for Fortification

Prior to fortification, no significant differences in Fe concentration existed among product types (70–73 ppm Fe) (Figure 2b). After fortification with 200 ppm of Fe, significant differences in Fe concentration were observed among product types (Figure 2c). The highest Fe concentrations were observed in fortified unpolished split (196.7 ppm) and polished football (191.5 ppm) dal. Polished football dal, which is typically polished with water and/or vegetable oil after milling, performed best in the context of uniformity of mixing with the fortificant solution and drying in the shaker-when placed in the shaker, the polished football dal moved more and agitated more quickly in the mixing trays. This helped to distribute the heat over the surface of the dal, hence it dried more uniformly and did not stick to the tray surface when wet. Selection of dehulled lentil rather than whole lentil was important, because removal of the seed coat has a significant effect on reducing the levels of polyphenolic compounds, thereby increasing Fe bioavailability [21].

For commercial-scale fortification, any of the four lentil product types potentially could be fortified. Consumer demand and the relative cost and availability of the various processing techniques would be important considerations. Successful fortification to produce fortified food depends on the interactions among the food vehicle, fortificant and the fortification technique. Dehulled lentil dal is available in three colors—red, yellow and green. Red cotyledon lentil was selected for fortification since it is the most widely consumed form of lentil dal, with wide acceptability in South Asia and the Middle East [28]. Consumers from some countries in these regions consume lentil as an essential component of their typical daily diet. Yellow and green lentil dal samples also were fortified and no significant differences were observed for final Fe concentration when fortified with similar concentrations of Fe fortificants (data not shown). Hence, any of red, yellow or green lentil dal could be fortified with the Fe fortificants.

### 3.2. Selection and Evaluation of the Most Suitable Fe Fortificant for Lentil

The success of food fortification programs is based on the chemistry between food vehicles and the fortificant selected to fortify foods [29]. Different food vehicles may contain different moisture levels and oxidizing agents that can react with fortificants and develop rancidity, metallic taste, off-color or degradation of vitamins, all factors that can influence bioavailability [30,31].

NaFeEDTA was shown previously to be two to four times more effective for increasing absorption of dietary Fe in humans compared to FeSO_4_ and ferrous fumarate [32]. It also was reported that Fe absorption was increased by using a mixture of FeSO_4_ and NaFeEDTA, instead of NaFeEDTA alone [33]. In another study, NaFeEDTA was proven to be a promising cost effective, water-soluble and highly bioavailable Fe fortificant that improved the Fe status of Vietnamese woman when consumed for 6 months (10 mg Fe for 6 days/week) [33]. These authors also reported that the prevalence of Fe deficiency and Fe deficiency anemia were reduced from 62.5% to 32.8% and from 58.3% to 20.3%, respectively.

The effect of NaFeEDTA-fortified wheat flour on urinary zinc extraction was studied and no effect was found in children [34]. Another study revealed no significant negative effects of NaFeEDTA-fortified bread (bread made with 100 g of NaFeEDTA-fortified wheat flour that contained 5 mg of Fe and was consumed as a single meal per day) consumption on Zn and Ca metabolism, and that NaFeEDTA might increase Zn absorption and Fe bioavailability from the low bioavailability diets [35]. In another study, NaFeEDTA was shown to have no influence on absorption or urinary excretion of Mn [36]. NaFeEDTA-fortified fish sauces also increased significantly the amounts of Hb and serum ferritin when provided to iron-deficient, anemic school children in Cambodia [37].

The review of the safety and efficacy of different dietary strategies for improving Fe status revealed that there are no reported data that demonstrate specific adverse effects of iron-fortified food items [38]. Moreover, the daily dose of Fe is much lower from fortified food than on supplementation [39]. The joint FAO/WHO Expert Committee on Food Additives (JECFA) summarized data on the basis of acute and chronic toxicity, reproduction, carcinogenicity, genotoxicity and teratogenicity of EDTA and its salts, such as NaFeEDTA [40]. The Committee also evaluated biochemical and toxicological aspects of using NaFeEDTA as a fortificant and stated that: (i) Fe from NaFeEDTA is released from the chelate to the common non-heme iron pool before Fe absorption; (ii) a very small fraction (1–2%) of NaFeEDTA is absorbed intact and is rapidly and completely excreted via the kidneys in the urine; (iii) dietary Fe fortification with NaFeEDTA does not increase the risk of iron accumulation in iron-replete individuals, and has no negative influence on the absorption of other micronutrients, such as Zn; and (iv) NaFeEDTA has low oral toxicity and does not induce gene mutations when tested with bacterial and mammalian cells in vitro. In addition, considering the cost of fortificant, NaFeEDTA is more expensive compared to FeSO_4_·7H_2_O and FeSO_4_·H_2_O, but its extra cost can be offset by its higher bioavailability in phytate-rich foods such as lentil [14].

### 3.3. Selection of Appropriate Methods for Fortification

#### 3.3.1. Techniques Used for Lentil Fortification

**Figure 3:**
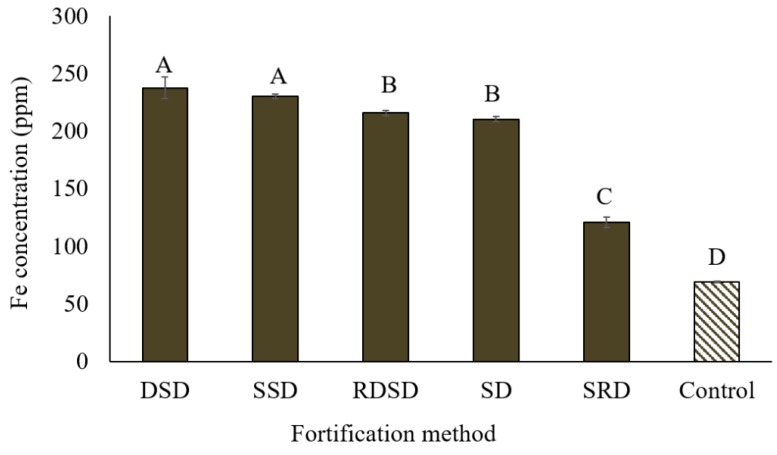
Iron concentration in polished football lentil dal fortified with FeSO_4_·7H_2_O solution (1600 ppm Fe) at 10 mL/100 g lentil dal using five different techniques. DSD = lentil dal oven dried for 10 minutes followed by soaking in fortificant solution and drying at 80 °C; SSD = lentil dal sprayed with fortificant solution followed by shaking and drying; RDSD = lentil dal rinsed, oven dried, followed by soaking in fortificant solution and then drying; SD = lentil dal directly soaked in fortificant solution followed by drying; SRD = lentil dal soaked in fortificant solution followed by rinsing with deionized water and drying. Different letters within the figure represent significant differences (*p* < 0.05).

**Figure 4:**
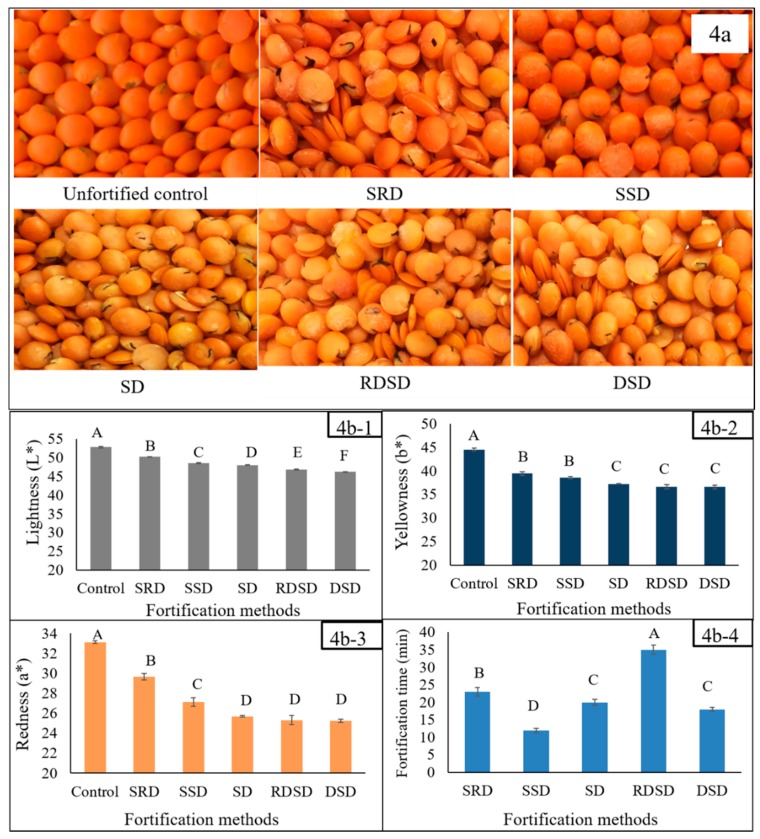
(**a**) Fe-fortified lentil developed by five different fortification methods: SRD = lentil dal soaked in fortificant solution followed by rinsing with deionized water and drying; SSD = lentil dal sprayed with fortificant solution followed by shaking and drying; SD = lentil dal directly soaked in fortificant solution followed by drying; RDSD = lentil dal rinsed, oven dried, followed by soaking in fortificant solution and then drying; DSD = lentil dal oven dried for 10 minutes followed by soaking in fortificant solution and drying at 80 °C; (**b1**–**b4**) Effect of different fortification methods on changes in lightness (L*), yellowness (b*) and redness (a*) score of Fe-fortified lentil dal and on the fortification process. Different letters within each figure represent significant differences (*p* < 0.05).

Significant variation in Fe concentration was found among the five methods used to fortify lentil dal. The highest concentrations of Fe were found with the DSD (lentil dal oven dried, soaked, followed by oven drying) and SSD (lentil dal sprayed with fortificant solution followed by shaking and drying) methods (Figure 3). Although the highest Fe absorption into the lentil seed was observed with DSD, the discoloration (increased darkness) of the final product may cause concern in the context of expected consumer preferences and longer fortification time (Figure 4). The homogeneity of Fe concentration was tested by randomly selecting six samples from the mixing tray. All samples contained similar amounts of Fe (215–220 ppm) after fortification.

#### 3.3.2. HunterLab Colorimetric Measurements of Fe-Fortified Lentil Samples

The HunterLab results indicated significant variation for all three scales (L*, a* and b*), indicating off-color development due to fortification (Figure 4b-1–b-3). The highest values for all three scales were found for the unfortified control lentil dal sample. The lowest L* value was found for the DSD sample, whereas the lowest a* and b* values were found for the samples produced by the SD, RDSD and DSD methods. The L*, a* and b* values ranged from 46.3 to 52.8, 25.3 to 33.1 and 36.6 to 44.6, respectively. The shortest processing time was required with the SSD method (Figure 4b-4), which also generated off- color but significantly less compared to the SD, RDSD and DSD methods.

#### 3.3.3. Assessment of Appropriate Temperature and Duration for Drying Fortified Lentil Dal

Temperature has been shown to have a significant effect on the drying time required to achieve a level of moisture suitable for safe storage [41]. The results from the assessment of appropriate temperature and duration for drying fortified lentil dal showed that with an increase in temperature caused by raising the light bulb wattage, there was an increase in the temperature (°C) of both the aluminum foil tray used for fortification and the fortified lentil seed. An inverse relationship was observed between total drying time and temperature (Figure S1). The temperature used to dry fortified lentil dal should be optimized to avoid off-color development, as a relationship between temperature and off-color development in fortified foods has been observed [30]. Using the 250-watt bulb, the temperature rose to 75 °C, which dried the fortified lentil dal in the shortest time (12–14 min). The moisture content of the fortified dal was approximately 14%, which is similar to the moisture content (%) of dehulled lentil dal (13–14)% that is commercially available in the local market [42]. During fortification, lentil dal was treated with fortificant solution and then heat was applied to dry the product. This process might reduce the level of phytate and phenolics level to some extent, and enhance the bioavailability of both Fe and Zn [43].

### 3.4. Assessment of the pH of Solutions Prepared with Three Fe Fortificants over a Range of Concentrations

**Figure 5:**
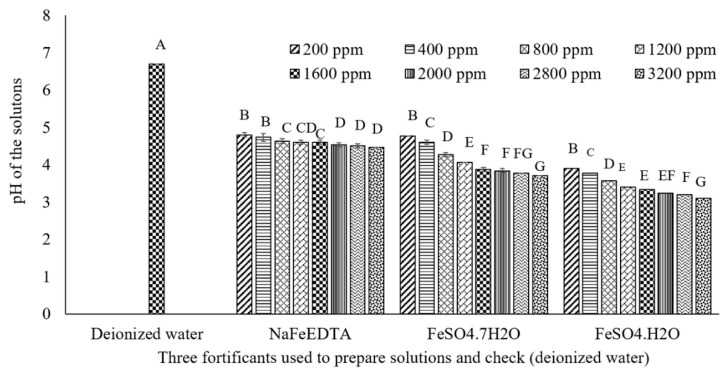
pH of Fe solutions prepared with three fortificants (NaFeEDTA, FeSO_4_·7H_2_O, and FeSO_4_·H_2_O) ranging in concentration from 200 to 3200 ppm. Different letters within each figure represent significant differences (*p* < 0.05).

Measurement of pH over a range of concentrations of the Fe fortificants showed an inverse relationship between pH and an increase in the concentration of Fe in the solution. The pHs of the three fortificant solutions were lower (<5) than that of deionized water (6.7). The rate of decrease of pH with an increase in Fe concentration was highest for FeSO_4_·H_2_O, followed by FeSO_4_·7H_2_O and NaFeEDTA (Figure 5). The pH of the fortificant solution would have an effect on the solubility of Fe [44]. Both pH and redox potential influence the oxidation state of Fe, and both the Fe^+2^ and the Fe^+3^ form are used for fortification. Both have unfilled orbits that can react with electron-rich components, thus influencing organoleptic attributes and bioavailability [45]. The oxidation-reduction reactions (redox potential) in fortified foods, due to the addition of Fe that can react with phenolic compounds, cause off-color development [43]. Ferrous ion oxidizes to the ferric form as redox potential increases, but remains constant at a lower redox potential [30,44]. The solubility of FeSO_4_ in 0.1 M HCI was reported to decrease by 74% with changes in pH over the range of 2–6, but remained constant for NaFeEDTA [45]. In this study, an increase in FeSO_4_ concentration resulted in a faster rate of pH reduction in comparison to NaFeEDTA. Moreover, to obtain a similar amount of soluble Fe at a specific pH, more FeSO_4_ is required than NaFeEDTA. This may cause a major change in the organoleptic characteristics of lentil dal. This study showed that NaFeEDTA would be a better choice than FeSO_4_ for fortification of lentil dal.

### 3.5. Estimation of Fe Concentration in Fortified Lentil Dal Samples Using F-AAS

The concentration of Fe in fortified lentil dal increased with an increase in Fe concentration in the fortificant solution (Table 1). Off-color development also increased gradually with an increase in the Fe concentration of the fortificant (Table 2).

### 3.6. Assessment of the Appropriate Dose of Fe 

Consideration of the appropriate dose of Fe is important for optimizing the amount of fortificant required to provide a major part of the estimated average requirement (EAR) for available Fe. The WHO has suggested suitable iron compounds to fortify specific food vehicles [13]. For instance, NaFeEDTA was suggested to fortify high extraction wheat flour, sugar, soy sauce, and fish at different rates. The bioavailability of Fe depends on the levels of various compounds present in the food vehicle, e.g., phytate, dietary fiber, tannins and other polyphenols [25,46]. These components can reduce the absorption of micronutrients, e.g., Fe, Zn. Moreover, Fe of plant origin is exclusively non-heme Fe, which is less bioavailable than the heme Fe from animal sources [46,47]. In this study, lentil dal fortified with three different fortificants showed an increase in Fe concentration with an increase in the Fe concentration in the fortificant solution. Lentil seed may exhibit a wide range in Fe concentration [7]. According to the FAO and WHO, EARs for iron having 10% bioavailability are 29.4 and 10.8 mg Fe day^−1^ for females and males, 19–50 years of age, respectively [13]. Therefore, 50 g of unfortified dehulled lentil could provide approximately 3.5 mg of Fe, based on the Fe concentration in the control lentil dal sample. The bioavailability may decrease if the dal is prepared with spices or condiments and is eaten with other foods such as rice, bread or vegetables, which may contain phytate, polyphenols or other components that reduce the absorption of Fe. To obtain a major portion of daily Fe from food fortificants, an optimum dose should be recommended. In this study, it was shown that lentil dal fortified with 1600 ppm of Fe could provide approximately 130–140 ppm of Fe per 100 g of lentil. Therefore, 50 g of fortified lentil could provide approximately 10 mg of Fe (6.5–7 mg of Fe from the fortificant + 3.5 mg from the lentil). This could provide a major portion of the EAR. Currently, 30–45 mg kg^−1^ ferrous sulphate and 250 mg kg^−1^ NaFeEDTA are used to fortify wheat flour and soy/fish sauce, respectively [13].

### 3.7. HunterLab Colorimeter Measurements of Stored Fe-Fortified Dal Samples 

**Figure 6:**
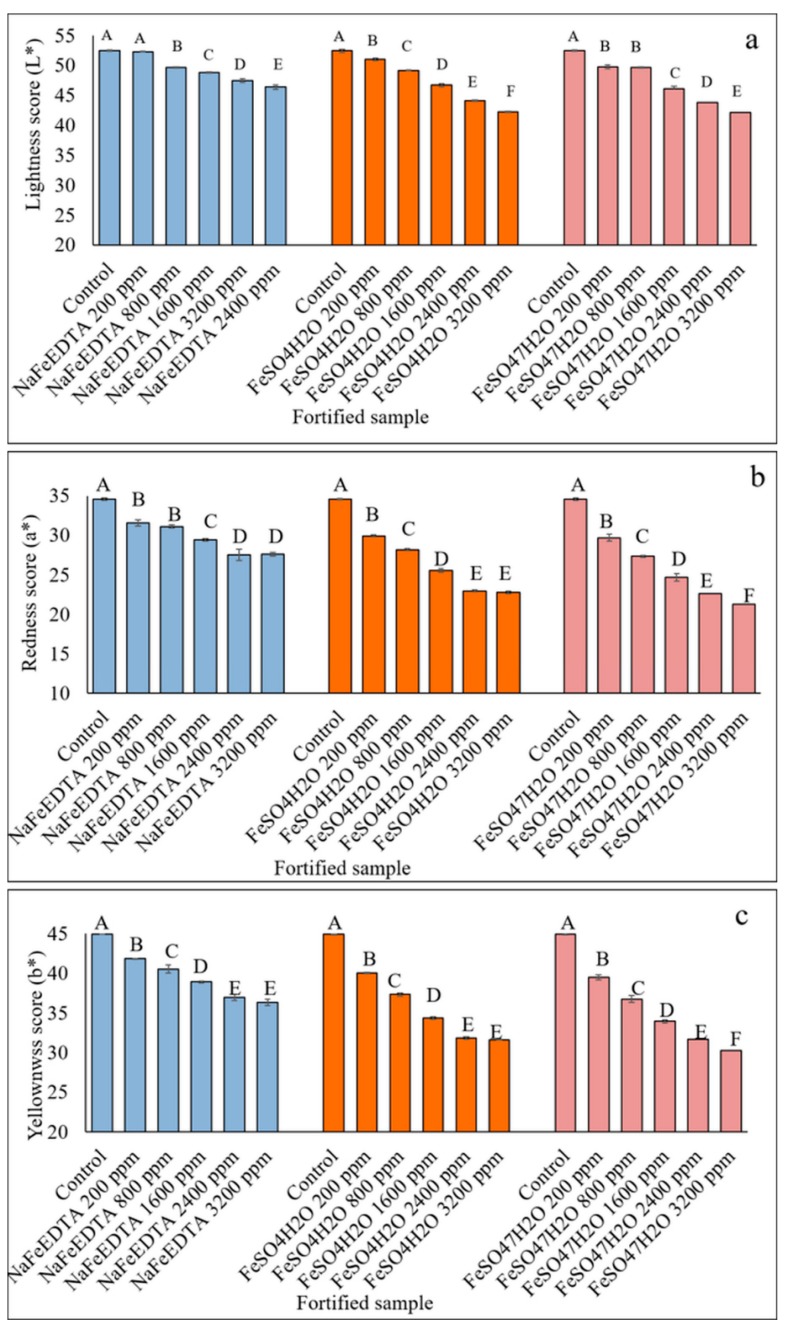
Effect of increasing Fe concentration on lightness (L*), redness (a*) and yellowness scores (b*) of lentil dal samples fortified with FeSO_4_·7H_2_O, NaFeEDTA and FeSO_4_·H_2_O at five different concentrations ranging from 200 to 3200 ppm. Different letters within each figure represent significant differences (*p* < 0.05).

Color attributes influence the acceptability of a food product to consumers. The L*, a* and b* scores were significantly decreased with an increase in Fe concentration provided by any of the fortificants. Significant variation in color was observed among lentil dal samples fortified with the three fortificants at any concentration. Samples fortified with NaFeEDTA had higher L*, a* and b* scores, similar to those of the control, indicating less off-color development when compared to dal samples fortified with FeSO_4_·7H_2_O or FeSO_4_·H_2_O (Figure 6).

**Figure 7:**
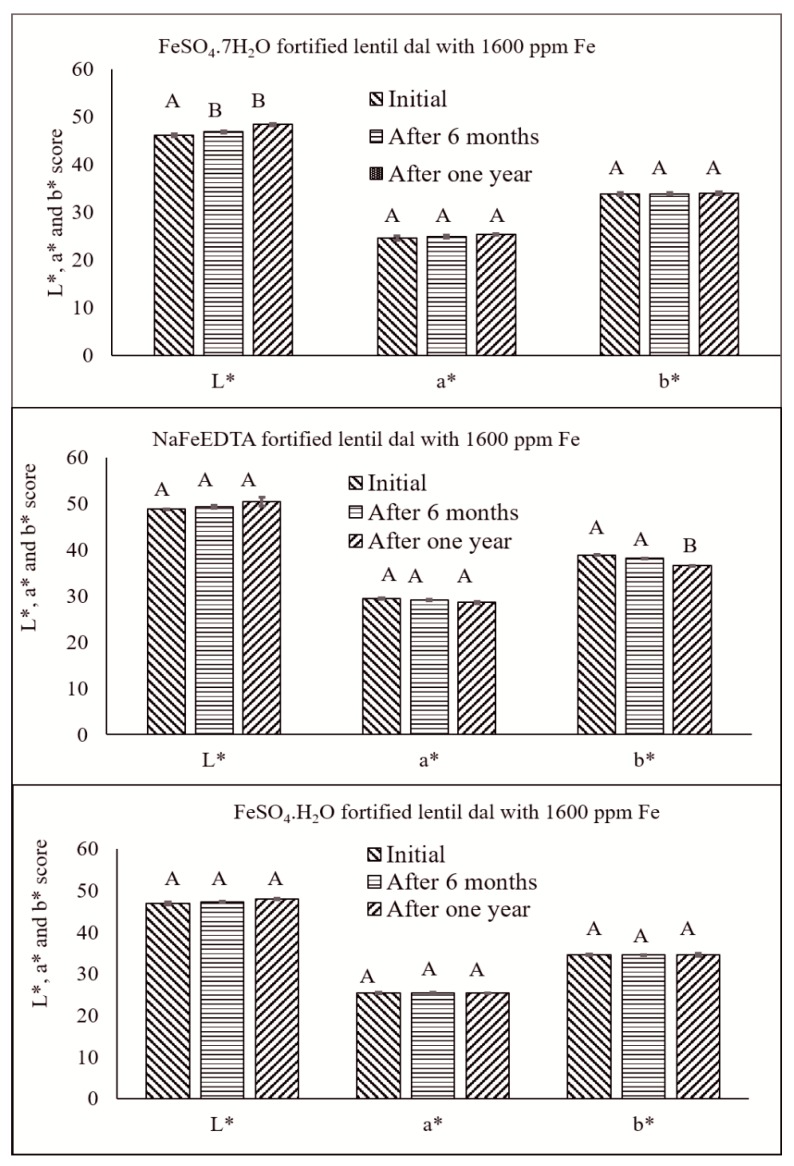
Effect of storage time on changes in L*, a* and b* score of football lentil samples fortified with 1600 ppm of Fe using FeSO_4_·7H_2_O, NaFeEDTA and FeSO_4_·H_2_O. Different letters within each figure represent significant differences (*p* < 0.05).

The usual expectation for any Fe-fortified food product is that it does not exhibit any off-color. The dark color of the micropylar area of fortified lentil dal possibly could be used as an indicator to help consumers distinguish between fortified and unfortified lentil dal, where the micropylar region is white. The L*, a* and b* color values for the fortified lentil dal samples showed some inverse relationships with the progress of storage time (Table 2). Lightness (L*) increased slightly, but a* and b* decreased in all of the fortified lentil dal samples over time. Initially, just after fortification, the L* value ranged from 50.6 (unfortified control) to 42.2 (fortified with 3200 ppm of FeSO_4_·7H_2_O), which was similar to the samples fortified with FeSO_4_·H_2_O (42.6). The range was narrower for the L* value of samples fortified with NaFeEDTA (50.6 to 46.4) (Table 2). For all three fortificants, after 6 months and one year of storage of fortified lentil dal, there was an increasing trend in L*, but a decreasing trend for the a* and b* values (Table 2). The non-significant differences in the L*, a* and b* scores for the unfortified and fortified lentil samples provides assurance that the minor changes observed will not influence consumer acceptability. The L*, a* and b* values for fortified lentil dal, prepared with the three fortificants at 1600 ppm of Fe, showed numerical decreases, but these were not significant for the three storage periods, except for the L* and b* scores for the FeSO_4_·7H_2_O-fortified and the NaFeEDTA-fortified samples, respectively (Figure 7). These small changes may be caused by the presence of very small amounts of lipid (1.52–2.95%) [48] that could increase the likelihood of lipid oxidation and result in off-color development over time.

### 3.8. Boiling Time Estimation of Fortified Samples Compared to the Unfortified Control

The boiling time of lentil dal is important and may influence consumer acceptability due to energy and time consumption during cooking. Compared to unfortified lentil dal, the fortified lentil dal should take equal or less time to cook, and have similar texture, taste and appearance after cooking. Among the four samples that were cooked to determine the variability in boiling time, all had similar cooking times (Figure S2). Fortification had no significant influence on the boiling time of FeSO_4_·7H_2_O-, FeSO_4_·H_2_O- or NaFeEDTA-fortified samples compared to the control.

### 3.9. Iron Concentration, Relative Fe Bioavailability and Phytic Acid Concentration of Fortified Lentils

Significant differences were observed among fortified and unfortified lentil samples in Fe concentration, relative Fe bioavailability and phytic acid concentration (Table 3). Similar iron and phytic acid concentrations were observed in FeSO_4_·7H_2_O- and NaFeEDTA- fortified samples. The unfortified lentil samples were statistically different than the three fortified samples for all four measurements. The relative bioavailability was similar for all three fortified lentil dal samples. Iron concentration and relative Fe bioavailability ranged from 68.7 to 238.5 ppm and 68.3 to 104.9, respectively. The relative Fe bioavailability of the three cooked fortified lentil dal samples was 1.4 to 1.5 times higher than that of unfortified cooked lentil sample (control). Phytic acid concentration ranged from 7.2 to 8.0 mg g^−1^.

Fortification of lentil dal is more complex than fortifying flour, beverages and most other food products due to the requirement to apply fortificant solution to the surface of the dal. Considering all of the results from the various experiments, it was concluded that lentil dal could be used as a vehicle for Fe fortification and that NaFeEDTA was the most suitable Fe fortificant for lentil dal. These results represent baseline data for the commercial production of Fe-fortified lentil dal. This research is unique in the context of lentil dal fortification, and will be followed by sensory evaluation to select the most appropriate fortificant after evaluation of overall acceptability. Results from sensory evaluation with both uncooked and cooked fortified lentil dal compared favorably with the control and will be described in a subsequent manuscript. Community-based efficacy and effectiveness studies with fortified lentil in the target populations will be required. The bioavailability of fortified lentil in a large-scale human trial also could be evaluated to obtain an empirical estimate of the amount of Fe required to provide a major portion of the EARs for Fe in regions where Fe deficiency exists.

## Figures and Tables

**Table 1:** Fe concentration (ppm) in polished football lentil dal samples prepared using three fortificants (FeSO_4_·7H_2_O, NaFeEDTA and FeSO_4_·H_2_O) at concentrations ranging from 100 to 3200 ppm.

Fe Concentration in Fortificant Solution (ppm)	Fe Concentration in Fortified Lentil Dal		
	FeSO_4_·7H_2_O	NaFeEDTA	FeSO_4_·H_2_O
Control	69.0 ± 0.9 ^a^	69.0 ± 0.9 ^a^	65.6 ± 0.8 ^a^
100	76.0 ± 1.9 ^a^	83.7 ± 2.5 ^a^	71.8 ± 0.7 ^b^
400	132.5 ± 3.2 ^b^	113.2 ± 4.2 ^b^	108.6 ± 1.1 ^c^
800	147.9 ± 4.7 ^c^	182.9 ± 5.8 ^c^	151.4 ± 2.8 ^d^
1200	157.8 ± 4.3 ^c^	185.3 ± 5.6 ^c^	185.0 ± 6.6 ^e^
1600	203.6 ± 3.9 ^d^	205.3 ± 2.8 ^d^	207.5 ± 3.9 ^f^
2000	217.5 ± 8.2 ^d^	274.7 ± 5.6 ^e^	261.8 ± 3.9 ^g^
2400	246.6 ± 9.3 ^e^	309.7 ± 10.0 ^f^	322.3 ± 3.7 ^h^
2800	286.7 ± 6.0 ^f^	346.7 ± 5.2 ^g^	363.5 ± 6.2 ^i^
3200	349.0 ± 1.8 ^g^	326 ± 3.1 ^h^	381.7 ± 3.6 ^j^

*^a^* Mean ± SD. Mean scores for Fe concentration followed by different letters within columns are significantly different (*p* < 0.001).

**Table 2:** Lightness (L*), redness (a*) and yellowness (b*) scores of fortified lentil samples prepared using FeSO_4_·7H_2_O, NaFeEDTA and FeSO_4_·H_2_O at concentrations ranging from 100 to 3200 ppm after six months and after one year of storage.

Fe Concentration (ppm)	Lightness (L*)			Redness (a*)			Yellowness (b*)		
	Initial	After 6 Months	After One Year	Initial	After 6 Months	After One Year	Initial	After 6 Months	After One Year
FeSO_4_·7H_2_O fortified samples									
Control	50.6 ± 0.4 ^a^	50.8 ± 0.2 ^a^	51.0 ± 0.2 ^a^	31.5 ± 0.2 ^a^	31.3 ± 0.2 ^a^	30.6 ± 0.6 ^a^	41.6 ± 1.0 ^a^	41.2 ± 1.0 ^a^	40.3 ± 1.0 ^a^
200	49.9 ± 0.6 ^ab^	50.6 ± 0.6 ^a^	52.0 ± 0.5 ^b^	29.7 ± 0.8 ^b^	29.4 ± 0.8 ^b^	28.8 ± 0.8 ^b^	40.5 ± 0.1 ^b^	38.9 ± 0.1 ^b^	37.9 ± 0.1 ^b^
800	49.6 ± 0.2 ^b^	50.3 ± 0.1 ^a^	51.5 ± 0.0 ^b^	27.4 ± 0.3 ^c^	26.8 ± 0.2 ^c^	25.8 ± 0.3 ^ac^	37.8 ± 0.3 ^c^	36.4 ± 0.3 ^c^	34.6 ± 0.3 ^c^
1600	46.2 ± 0.5 ^c^	46.9 ± 0.5 ^b^	48.5 ± 0.4 ^c^	24.6 ± 0.7 d	24.9 ± 0.6 ^d^	25.5 ± 1.2 ^c^	36.4 ± 0.1 ^d^	33.9 ± 0.1 ^d^	34.0 ± 0.1 ^c^
2400	43.9 ± 0.2 ^d^	44.5 ± 0.1 ^c^	45.8 ± 0.2 ^c^	22.6 ± 0.2 ^e^	22.2 ± 0.1 ^e^	21.3 ± 0.2 ^d^	32.0 ± 0.3 ^e^	31.2 ± 0.1 ^e^	30.0 ± 0.4 ^d^
3200	42.1 ±0.6 ^e^	42.7 ± 0.6 ^d^	43.9 ± 0.6 ^d^	21.3 ± 0.8 ^f^	34.4 ± 0.9 ^f^	20.3 ± 1.2 ^d^	30.0 ± 0.2 ^f^	29.7 ± 0.7 ^f^	28.6 ± 0.3 ^e^
NaFeEDTA fortified samples									
Control	50.5 ± 0.4 ^a^	50.8 ± 0.2 ^a^	50.8 ± 0.2 ^a^	31.5 ± 0.2 ^a^	31.3 ± 0.3 ^a^	30.6 ± 0.6 ^a^	41.6 ± 0.3 ^a^	41.2 ± 0.1 ^a^	40.3 ± 0.7 ^a^
200	50.4 ± 0.1 ^a^	51.0 ± 0.2 ^a^	51.0 ± 0.2 ^a^	31.6 ± 0.7 ^a^	31.1 ± 0.8 ^a^	30.3 ± 0.8 ^a^	41.9 ± 0.1 ^a^	41.5 ± 0.1 ^a^	40.6 ± 0.3 ^a^
800	50.1 ± 0.2 ^a^	50.6 ± 0.6 ^a^	50.6 ± 0.6 ^b^	31.1 ± 0.3 ^a^	30.5 ± 0.2 ^a^	29.0 ± 0.5 ^b^	40.6 ± 0.9 ^b^	39.3 ± 0.4 ^a^	36.9 ± 0.8 ^b^
1600	48.8 ± 0.1 ^b^	52.0 ± 0.5 ^b^	52.0 ± 0.5 ^b^	29.4 ± 0.3 ^b^	29.1 ± 0.2 ^b^	28.6 ± 0.4 ^b^	38.9 ± 0.2 ^c^	38.2 ± 0.2 ^b^	36.6 ± 0.5 ^b^
2400	47.5 ± 0.2 ^c^	50.3 ± 0.1 ^c^	50.3 ± 0.1 ^c^	27.5 ± 1.3 ^c^	27.0 ± 1.2 ^c^	26.1 ± 1.1 ^c^	36.3 ± 0.7 ^d^	35.8 ± 0.6 ^c^	34.6 ± 0.6 ^c^
3200	46.4 ± 0.5 ^d^	51.5 ± 0.0 ^d^	51.5 ± 0.0 ^c^	27.8 ± 0.4 ^c^	27.4 ± 0.4 ^c^	26.5 ± 0.4 ^c^	36.9 ± 0.7 ^d^	36.4 ± 0.8 ^c^	35.2 ± 0.9 ^c^
FeSO_4_·H_2_O fortified samples									
Control	50.5 ± 0.4 ^a^	50.5 ± 0.4 ^a^	50.8 ± 0.2 ^a^	51.2 ± 0.3 ^a^	31.5 ± 0.2 ^a^	31.3 ± 0.2 ^a^	30.6 ± 0.6 ^a^	41.6 ± 0.3 ^a^	41.2 ± 0.1 ^a^
200	51.1 ± 0.5 ^a^	51.1 ± 0.5 ^a^	51.3 ± 0.3 ^b^	51.7 ± 0.3 ^b^	30.0 ± 0.7 ^b^	29.9 ± 0.7 ^a^	29.8 ± 0.7 ^b^	39.9 ± 0.1 ^b^	39.6 ± 0.1 ^b^
800	49.3±0.7 ^b^	49.7 ± 0.7 ^b^	50.4 ± 0.5 ^b^	27.9 ± 0.3 ^c^	27.6 ± 0.4 ^c^	27.1 ± 0.4 ^a^	37.3 ± 0.9 ^c^	36.9 ± 0.4 ^c^	36.5 ± 0.8 ^c^
1600	46.9 ± 0.7 ^c^	47.3 ± 0.4 ^c^	48.1 ± 0.2 ^c^	25.4 ± 0.3 ^d^	25.4 ± 0.3 ^d^	25.4 ± 0.4 ^c^	34.6 ± 0.2 ^d^	34.6 ± 0.2 ^d^	34.6 ± 0.5 ^d^
2400	44.4 ± 0.6 ^d^	44.7 ± 0.4 ^d^	45.4 ± 0.4 ^d^	23.3 ± 0.7 ^e^	22.8 ± 0.7 ^e^	21.9 ± 0.9 ^d^	32.2 ± 0.7 ^e^	31.9 ± 0.6 ^e^	30.2 ± 0.6 ^e^
3200	42.6 ± 0.3 ^e^	42.6 ± 0.3 ^e^	42.7 ± 0.5 ^e^	22.7 ± 0.7 ^e^	22.1 ± 0.7 ^e^	21.1 ± 0.8 ^d^	31.5 ± 0.7 ^e^	30.9 ± 0.8 ^f^	29.8 ± 0.9 ^f^

*^a^* Mean ± SD. Mean scores for lightness (L*), redness (a*) and yellowness (b*) score followed by different letters within columns are significantly different (*p* < 0.001).

**Table 3:** Mean iron (Fe) concentration (ppm), relative bioavailability (ng ferritin (mg protein)^−1^) and phytic acid concentration (mg g^−1^) of four cooked freeze-dried lentil samples.

Cooked Lentil Sample	Fe Concentration (ppm) *^a^*	Ferritin Formation (ng Ferritin (mg Protein)^−1^) *^a^*	Relative Fe Bioavailability (% Control Lentil) *^a^*	Phytic Acid (mg g^−1^) *^a^*
Unfortified dehulled lentil	68.7 ± 0.3 ^a^	12.7 ± 1.0 ^a^	68.3 ± 14.8 ^a^	8.0 ± 0.1 ^a^
NaFeEDTA fortified (1600 ppm Fe)	230.8 ± 8.5 ^b^	17.4 ± 2.7 ^b^	100.5 ± 7.5 ^b^	8.0 ± 0.2 ^a^
FeSO_4_·H_2_O fortified (1600 ppm Fe)	220.5 ± 2.1 ^c^	17.6 ± 2.2 ^b^	104.9 ± 16.7 ^b^	7.2 ± 0.1 ^c^
FeSO_4_·7H_2_O fortified (1600 ppm Fe)	238.5 ± 4.7 ^b^	21.2 ± 1.9 ^b^	103.4 ± 10.4 ^b^	7.4 ± 0.1 ^b^

*^a^* Mean ± SD. Mean scores for Fe concentration, bioavailability (ng ferritin (mg protein)^−1^), relative Fe bioavailability (% control lentil) and phytic acid (mg g^−1^) followed by different letters within columns are significantly different (*p* < 0.001).

## Supplementary Materials

The following are available online at www.mdpi.com/2072-6643/9/8/863/s1, Figure S1: Effect of increasing light bulb wattage on temperature (°C) and drying time (min) of fortified lentil samples, Figure S2: Effect of the three fortificants solution used to prepare three fortified lentil samples (FeSO_4_·7H_2_O, NaFeEDTA and FeSO_4_·H_2_O) on boiling time compared with one unfortified control sample. Different letters within each figure are significantly different (*p* < 0.05).
