# Supplementary figures and images for: Iron Fortification of Lentil (Lens culinaris Medik.) to Address Iron Deficiency
Source: Nutrients. 2017 Aug 11;9(8):863. doi: 10.3390/nu9080863 (PMC5579656; doi:10.3390/nu9080863)

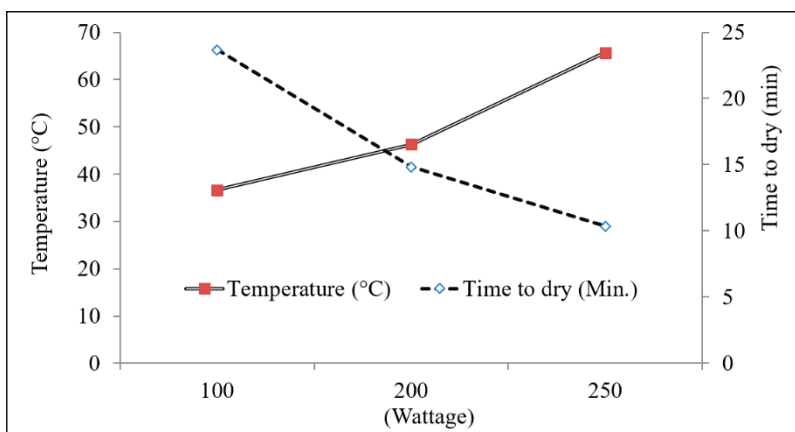

Supplementary Figure S1

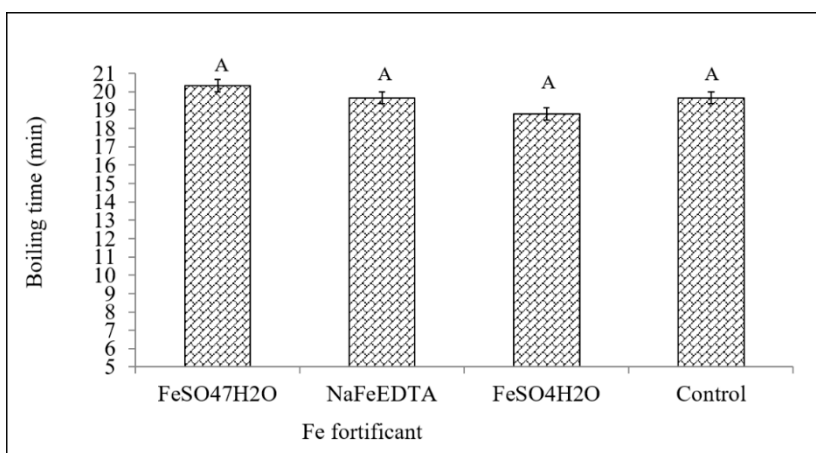

Supplementary Figure S2

Supplement: Supplementary file 1 [file nutrients-09-00863-s001.zip › nutrients-209970-supplementary.pdf]
